# Consensus Statement on Digital Health and Attention-Deficit/Hyperactivity Disorder by the European Network for ADHD (EUNETHYDIS): Modified Delphi Study

**DOI:** 10.2196/85638

**Published:** 2026-07-16

**Authors:** Anna Price, Emi Furukawa, Alessio Bellato, Pascal-M Aggensteiner, Matthew Bellringer, Claire Cattel, Samuele Cortese, David Daley, Manfred Döpfner, Federica Donno, Madeleine J Groom, Anna Kaiser, Christina Kini-Seery, Katarzyna Kostyrka-Allchorne, Jonna Kuntsi, Stuart Kyle, Elizabeth B Liddle, Giorgia Michelini, Emilie S Nordby, Nicholas Peres, Tashinga Ruzive, Anita Salimi, Douglas Sjöwall, Edmund J S Sonuga-Barke, James Wenger, Tamsin Newlove-Delgado

**Affiliations:** 1 Faculty of Health and Life Sciences, University of Exeter, Room 2.05, South Cloisters, University of Exeter St Luke's Campus Heavitree Road, Exeter, EX1 2LU, United Kingdom, 44 01392 726026; 2 Human Developmental Neurobiology Unit, Okinawa Institute of Science and Technology Graduate University, Okinawa, Okinawa, Japan; 3 School of Psychology, University of Southampton, Southampton, United Kingdom; 4 Central Institute of Mental Health, Department of Child and Adolescent Psychiatry and Psychotherapy, Mannheim, Germany; 5 Clinical Psychology of Childhood and Adolescence, Institute of Psychology, Friedrich Schiller University Jena, Jena, Germany; 6 BCS, The Chartered Institute for IT, Swindon, United Kingdom; 7 Oxford Health NHS, Oxford, United Kingdom; 8 Department of Psychology, Nottingham Trent University, Nottingham, United Kingdom; 9 Department of Psychiatry and Psychotherapy of Childhood and Adolescence, Faculty of Medicine, University of Cologne, Cologne, North Rhine-Westphalia, Germany; 10 Department of Biomedical Sciences, Neuroscience and Clinical Pharmacology, University of Cagliari, Cagliari, Sardinia, Italy; 11 Institute of Mental Health, University of Nottingham, Nottingham, United Kingdom; 12 German Center for Mental Health (DZPG), Partner Site Mannheim-Heidelberg-Ulm, Germany; 13 School of Psychology, University College Dublin, Dublin, Leinster, Ireland; 14 Department of Psychology, School of Biological and Behavioural Sciences, Queen Mary University of London, London, United Kingdom; 15 Social, Genetic and Developmental Psychiatry (SGDP) Centre, Institute of Psychiatry, Psychology and Neuroscience, King's College London, London, United Kingdom; 16 Llansamlet Surgery, Swansea, United Kingdom; 17 Division of Psychiatry, Haukeland University Hospital, Bergen, Vestland, Norway; 18 Torbay and South Devon NHS Foundation Trust, Torquay, England, United Kingdom; 19 University of Exeter, Exeter, United Kingdom; 20 Department of Clinical Neuroscience, Karolinska Institutet, Sweden, Stockholm, Sweden; 21 Institute of Psychiatry, Psychology and Neuroscience, King's College London, London, United Kingdom; 22 Department of Child and Adolescent Psychiatry, Institute of Psychiatry, Psychology and Neuroscience, King’s College London, , London, United Kingdom

**Keywords:** digital health, attention-deficit/hyperactivity disorder, ADHD, consensus, Delphi, European Network for ADHD, EUNETHYDIS

## Abstract

**Background:**

Digital technologies are becoming an important part of health care, including for individuals with attention-deficit/hyperactivity disorder (ADHD). Digital health innovations present valuable opportunities to provide flexible and tailored support for their diverse needs, along with significant challenges. Attentional, organizational, and motivational characteristics associated with ADHD may affect how individuals engage with digital tools. Potential risks include additional access barriers, the exclusion of underserved groups, and diminished quality of care. To help reduce these risks, the development, evaluation, and implementation of digital tools must be person-centered and guided by a comprehensive understanding of the diverse needs of all stakeholders.

**Objective:**

To advance research in this area, a multidisciplinary panel of ADHD specialists, technology experts, and individuals with lived experience of ADHD was formed. The panel worked together to agree on key priorities and considerations for developing, evaluating, and implementing digital technologies for ADHD. The recommendations are designed to be shared with the wider research community and to guide innovations in ADHD digital health to improve care.

**Methods:**

A modified Delphi approach was used to develop consensus. Key statements were drafted, building on discussions held during the European Network for ADHD (EUNETHYDIS) Special Interest Group meeting in 2024. An expert panel that included additional key stakeholders was convened. Draft statements were shared with panel members via a 2-round Delphi survey and discussion meetings, with final statements coproduced by the panel. Insights from multiple perspectives were incorporated, and consensus agreement was sought. Refined statements were shared with EUNETHYDIS members for ratification. Panel members were invited to contribute as coauthors.

**Results:**

An expert panel of 28 members (21 EUNETHYDIS Special Interest Group members, 7 invited experts) coproduced 30 consensus statements on ADHD and digital health. Agreement ranged from 78.6% (22/28) to 100% (28/28) for the first round (19 statements) and from 92.6% (25/27) to 100% (27/27) for the second round (30 statements). Final statements covered 4 topic areas: Opportunities and Aspirations, Development and Evaluation, Implementation, and Risks and Unintended Consequences. These were ratified in September 2025 by the EUNETHYDIS.

**Conclusions:**

This consensus process provides the first comprehensive set of key considerations for digital health care for people with ADHD and demonstrates the feasibility of achieving expert agreement on complex, rapidly evolving topics, such as digital health. Future work should focus on translating these considerations into more specific and practical implementation frameworks, identifying priorities, and connecting them to real-life stories and empirical evidence.

## Introduction

### Background

Digital health care for people with attention-deficit/hyperactivity disorder (ADHD) is a rapidly evolving and highly heterogeneous field. It is becoming an increasingly important source of information and an element of care [1,2] in many settings, especially given the long wait time for accessing services. Current digital health innovations for ADHD span multiple domains including assessment tools (eg, computerized, virtual reality, physiological measurements) [3], intervention platforms (eg, apps, games, online resources) [4,5], remote monitoring systems and technologies (eg, wearable devices, smartphone-based measures) [6], and care delivery models (eg, telehealth, mobile health, digital therapeutics) [7,8]. Aligning such varied innovations to unified standards is difficult. Challenges consistent with the broader digital health field also persist, including interdisciplinary tensions between engineers, clinicians, and designers [9]; inconsistent evaluation methods and outcome measures [1]; concerns about privacy and digital equity [10]; and gaps in real-world evidence [11]. The evidence for the effectiveness of digital technologies in health care for ADHD is still limited; technological advancements outpacing research progress create gaps between innovation and evidence [11]. A coordinated approach is needed to facilitate robust progress in the challenging but highly promising area of ADHD digital health research.

For digital innovations to be effective, ethically sound, and meaningful, requirements include user-centered holistic design; a strong evidence base; and attention to privacy, equity, and clinical integration. Methodologies for codevelopment, evaluation, and implementation of digital health innovations are still being refined [9,12], with ongoing challenges around effective multidisciplinary working practices, appropriate evaluation methods, and inclusive and sustainable implementation. Consensus statements and expert guidance on the use of digital health technologies exist for mental health conditions in general [13-16]; however, their applications to ADHD-specific innovations need to be examined, along with unique considerations for supporting those living with ADHD.

Characteristics associated with ADHD include attentional, motivational, organizational, and executive function differences. Such features are likely to affect engagement with, and the use of, digital tools and platforms requiring careful attention to interface design and content delivery [17,18]. Diverse presentations of ADHD, including its high comorbidity rates with mood, anxiety, and substance use disorders [19], and complex care pathways, make the tailoring of comprehensive care challenging [20,21]. Those affected may also be at risk for providing and receiving misinformation on digital platforms [22]. At the same time, intense engagement in topics of interest [23], enthusiasm for technologies and gaming [24], and agile and innovative thinking [25] suggest a strong potential for digital health care, especially given the possibility of personalized and adaptive care [26]. Multifaceted care needs, including medication management, psychoeducation, and behavioral and emotional regulation, can potentially be well-supported by integrated digital resources [27,28].

However, significant barriers continue to limit the progress of digital health care for ADHD. Research groups are innovating at pace; however, each group often works independently, knowledge is not always shared effectively across groups, language use is inconsistent, and the criteria for evaluating such innovations remain unclear. The development and evaluation of digital health innovations have also historically neglected to include the perspectives of individuals with ADHD [29], likely limiting their engagement. Furthermore, younger children and adolescents may access digital health care indirectly through parents or other caregivers, or via education professionals, raising a further set of challenges to navigate in designing the technology.

### Objectives

This project aimed to establish consensus for developing, evaluating, and implementing digital health innovations for individuals with ADHD. Such consensus, including the perspectives of people with ADHD together with those of clinicians, researchers, and technology specialists, can provide guideposts to help ensure that innovations are scientifically rigorous and clinically meaningful. This consensus-building effort addresses calls for evidence-based frameworks and standardized approaches for new innovations, which have been identified as critical needs in advancing care for ADHD [20,30].

We intend the consensus statements to provide a broad overview applicable to a wide range of digital resources (eg, psychoeducational content, peer support networks, self-help materials), technologies (eg, wearable devices, smartphone apps, web-based platforms, AI-powered aids), and systems (eg, electronic health records, telehealth infrastructures, virtual reality platforms, digital therapeutics ecosystems) used in the assessment, monitoring, and intervention for ADHD-related challenges [2,31]. Our statements are intended to complement and promote more focused reviews (eg, [32]), statements (eg, [33]), and practical guidelines (eg, [3]).

## Methods

### Overview

We used a modified Delphi approach to develop consensus. The process comprised three main phases: (1) initial statement development based on in-person discussions held during a European Network for ADHD (EUNETHYDIS) Special Interest Group (SIG) meeting and a review of the literature, (2) establishment of the expert panel and structured consensus-building through online surveys and virtual meetings, and (3) ratification by the EUNETHYDIS members. This process was intended to establish an initial, broad consensus among those working to improve digital health care for people with ADHD.

### Ethical Considerations

This research was conducted in line with the UK Standards for Public Involvement in Research [34], which include clear communication, working together, inclusive opportunities, impact, governance, and support and learning. Expert panel members (collaborators) were treated as equal partners during the codevelopment of statements and agreed to be named as contributors during each Delphi round. Formal ethical approval was not required; however, this research was conducted in line with international ethical guidelines [35].

### Participants

#### EUNETHYDIS

EUNETHYDIS is a European network of recognized ADHD researchers, which also includes affiliated members outside Europe [36]. At the annual EUNETHYDIS meeting in Cagliari (September 2024), a SIG was set up and chaired by AP, with the aim of building and disseminating evidence-informed consensus on developing and implementing digital health technologies for individuals with ADHD. Following in-person discussions, a consensus workstream—coordinated by AP, EF, and TN-D—was established, and the SIG members were invited to participate in the Delphi study (April-August 2025). Following the Delphi study, the consensus statements were shared with the EUNETHYDIS members via email, and the statements were ratified during the annual EUNETHYDIS meeting in Bonn (September 2025).

#### Public and Patient Involvement and Engagement

Public and patient involvement and engagement (PPIE) members were identified via the study networks of the lead authors and other SIG members. They were invited via email to participate in the Delphi study, with a plain accessible language summary of activities provided, and offered a meeting to answer any questions. They were provided with payments (vouchers) in recognition of their time, in line with UK involvement guidelines [34] and National Institute for Health and Care Research payment guidelines [37]. Following feedback during Discussion Meeting 2, PPIE members were offered an additional PPIE-focused meeting to aid communication.

#### Expert Panel

The expert panel for the Delphi study consisted of the SIG members and a selection of external stakeholders who were invited to provide a range of perspectives, including those of people with lived experience of ADHD (PPIE members) and 4 digital experts. While many of the EUNETHYDIS SIG members are also clinicians (mainly child psychiatrists and psychologists), an additional general practitioner was invited. The panel consisted of experts mostly working in Europe, considering we aimed to produce statements that would be applicable to the European geographical and health care context and recognizing the challenges of producing consensus statements appropriate to diverse settings globally. Panel members were provided with a plain language summary of the process, including practical information about the organizers, project remit, work involved (including that panel members would be named, with an opportunity for coauthorship), and a draft timeline (Multimedia Appendix 1).

### Delphi Survey Methodology

The two rounds of Delphi surveys were administered online via Qualtrics [38], with links to the survey sent via emails. For each draft statement, participants were asked to rate their agreement using a 4-point Likert scale (strongly agree, agree, disagree, and strongly disagree). We did not include a neutral choice, which can be difficult to interpret, but allowed participants to skip questions. Consensus was defined as a participant agreement level above 75% [39]. Responses of “strongly agree” and “agree” were defined as agreement with the statements.

For the first-round survey, participants were invited to provide comments and suggestions, regardless of their response choice, in a free-text box. As comments were generally short, and not made by all respondents to all questions, we did not undertake a formal process of coding these free-text responses. The results of the first-round survey were shared and discussed with panel members in an online meeting. Revisions were considered based on all the feedback obtained, even when the predetermined percentage of agreement for a statement had been reached. For the second-round survey, participants were asked to provide comments and suggestions only if they disagreed with the revised statements. A second consensus meeting was then held to discuss any final comments or minor modifications suggested. At this point, as all statements had reached consensus, only very minor changes were made (eg, changing word orders in a sentence). This resulted in a final set of statements, which were then ratified by the EUNETHYDIS members in September 2025.

## Results

### Overview

An expert panel of 28 members (21 EUNETHYDIS SIG members, 7 invited experts) coproduced 30 consensus statements on ADHD and digital health. The agreements ranged between 78.6% (22/28) and 100% (28/28) for the 19 statements in the first round and between 92.6% (25/27) and 100% (27/27) for the 30 statements in the second round. The final statements were ratified by EUNETHYDIS members in September 2025 and covered 4 topic areas: Opportunities and Aspirations, Development and Evaluation, Implementation, and Risks and Unintended Consequences. Consensus statements are presented in Textbox 1 and online (see section “ADHD and digital technology” [40]).

Textbox 1. **Consensus statements on digital health and attention-deficit/hyperactivity disorder (ADHD) from the modified Delphi study.**

**Section 1: Opportunities and Aspirations**

*Digital technologies have the potential to improve health services and support for people with attention-deficit/hyperactivity disorder (ADHD); however, there is a shortage of evidence-based tools that are suitable for clinical use. The statements in this section represent our consensus on some of the opportunities afforded by digital technologies, if they are rigorously and appropriately codeveloped, implemented, integrated, and evaluated. We acknowledge that there is a diversity of contexts, uses and users of digital technologies and that the evidence base for these potential benefits remains limited to date. Later in this statement, we indicate priorities to develop the evidence base in this area and provide further discussion of the potential risks and unintended consequences.*

 1. Digital technologies could improve timely access to assessment and evidence-based support for ADHD and related challenges.

 2. Digital technologies could enhance the flexibility and inclusivity of services by accommodating diverse preferences for content, access, language, stimulus types, and interactions.

 3. Digital technology could extend the reach of high-quality support for ADHD, especially given widespread challenges of limited mental health resources across countries with differing health infrastructure.

 4. Digital technology has the potential to provide just-in-time data to improve monitoring, feedback, and personalized support, as long as it is user-centered and addresses privacy concerns.

 5. Opportunities for improved provision must be viewed in the context of a range of risks and unintended consequences including, but not limited to, unregulated digital development, inappropriate substitution for clinical care, and widening of inequalities.

**Section 2: Development and Evaluation**

*Development processes for digital health technologies need to be agile and iterative and involve multiple stakeholders. Digital interventions are often complex interventions, and hence more work is needed to identify the necessary adaptations to ensure development and evaluation of digital interventions produce high-quality evidence to support their use. We recommend that methods development is a priority.*

**Part 1: Development**

 6. Digital health product development could be improved through better knowledge and resource sharing among researchers, clinicians, software engineers, and people with lived experience.

 7. User-centered development is key to ensure technologies are effective, by involving diverse stakeholders early, balancing researcher, clinician, and user priorities, and integrating continuous feedback.

 8. Researchers need to adopt multidisciplinary ways of working to ensure development integrates different perspectives, varied expertise, and a range of digital platforms and methodologies.

 9. Development of evidence-based digital technologies for ADHD requires support from institutions and funders to create and resource diverse, user-centered, and multidisciplinary teams.

 10. Digital product design needs to be sensitive to culture and context, as well as meeting clinical and privacy standards.

**Part 2: Evaluation**

 11. Digital health products should undergo rigorous evaluation to ensure their benefits and avoid harm.

 12. Guidelines and methodologies are needed to establish a reliable and robust evidence base, in the context of the rapid and iterative development of ADHD health technologies.

 13. The development of new technologies offers a potential opportunity for different forms of evaluation beyond traditional methodologies.

 14. It is important to assess effectiveness, usability, feasibility, and efficiency while incorporating large-scale, continuous real-world feedback.

 15. Evaluations must address inclusivity, reach, and impact on intended and unintended users.

 16. Objective and subjective outcome measures should be selected that are appropriate for use in digital environments, while considering and minimizing user burden.

 17. Adverse effects must be identified and evaluated, including consideration of the ways ADHD characteristics can impact attention, learning, motivation, and technology dependence.

 18. Research should report on user involvement and engagement, study design justification, and clinically meaningful outcomes including risks and unintended consequences.

**Section 3: Implementation**

*Priority statements for the implementation of digital health technologies for people with ADHD. We emphasize the importance of carefully considering the context of implementation, as well as the integration and interaction of digital and nondigital elements of care in complex health care systems. Training, support and infrastructure are also essential for the successful implementation and integration of these technologies, with further work needed on what specific “digital competence” might be needed for clinicians working with people with ADHD.*

 19. Integrate digital technology into comprehensive support for ADHD while recognizing its clinical, technical, evidence and equity limitations.

 20. Employ multidisciplinary and community-participatory strategies in order to make sure digital technologies are usable and useful in real-world settings.

 21. Those implementing digital technologies need to take responsibility for educating users about digital health products, their quality, and how to select suitable options.

 22. Clinicians working with people with ADHD require training and support to ensure they have the necessary competencies to use digital health technologies in their practice.

 23. Health and care services using digital technologies must have appropriate infrastructure and technical support in place to support end users, protect privacy, and avoid increasing the burden on clinicians or people with ADHD.

 24. Those implementing digital technologies must ensure transparency about data collection, privacy protections, and product limitations.

 25. There is a need for researchers and developers to consider sustainable implementation strategies including industry partnership, conducting economic evaluations, and planning for iterative development.

**Section 4: Risks and Unintended Consequences**

*We discuss the opportunities presented by digital technologies for people with ADHD, but we also recognize that there are risks and unintended consequences, which may be positive, negative, or mixed. This emphasizes the importance of careful and rigorous development, implementation and evaluation, and of paying attention to the ethical use of health technologies.*

 26. Digital health technologies for ADHD may be used in a range of ways and contexts, and therefore the risks and unintended consequences may be unpredictable, and specific to the user or users and the system.

 27. There are obvious risks relating to widening inequalities due to digital exclusion and other factors, and ways to monitor and mitigate this risk require further consideration.

 28. Care must remain person-centered. It must be recognized that not everyone benefits from or prefers digital tools and that some individuals with ADHD or with coexisting conditions may face challenges in interacting with digital technology.

 29. The use of digital technologies with children with ADHD and their families may also require specific consideration around issues and potential risks such as consent, privacy, and screen time.

 30. There is a risk that digital interventions may be used to replace rather than augment nondigital services, and hence we emphasize the importance of an integrated approach, and the provision of appropriate alternatives.

### Engagement

**Figure 1.**
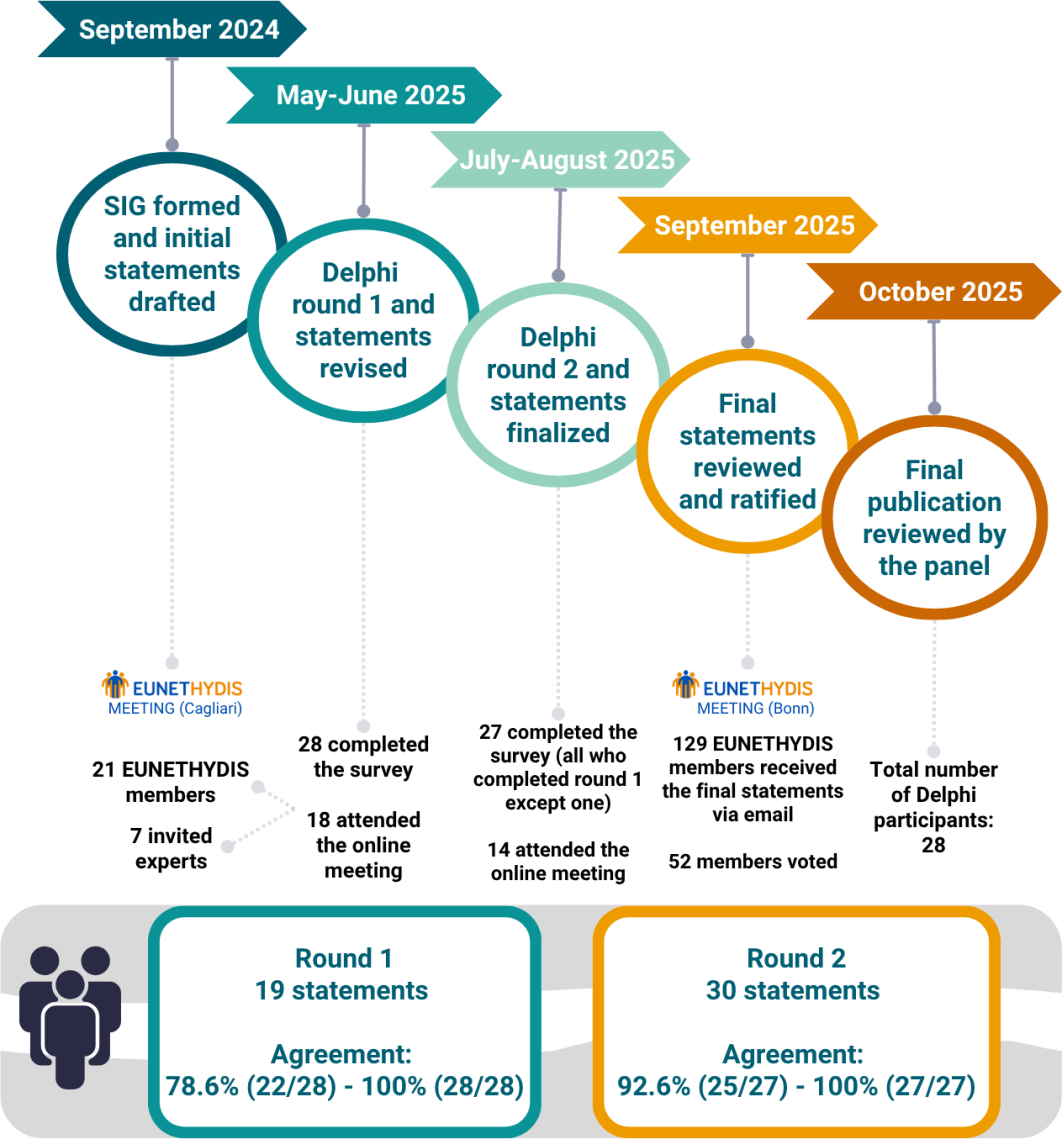
Flowchart of the modified Delphi study used to reach consensus on digital health and attention-deficit/hyperactivity disorder (ADHD), illustrating the recruitment of experts and the iterative process. EUNETHYDIS: European Network for ADHD; SIG: Special Interest Group.

Twenty-one EUNETHYDIS attendees participated in the initial SIG meeting in 2024 (Figure 1). An invitation to participate in the Delphi survey was sent to attendees and 7 additional identified experts. Twenty-eight respondents completed the round 1 survey, of whom 18 participated in the following online discussion meeting, and 27 completed the round 2 survey (1 participant who attended round 1 could not attend round 2). Fourteen participated in the second online discussion meeting. Final statements were shared with the EUNETHYDIS members (n=129) via email, with an opportunity for comment. The statements were then presented for ratification during the in-person members section of the EUNETHYDIS annual meeting (September 2025; n=52 attendees).

### Participant Characteristics

The expert panel of 28 collaborators consisted of ADHD researchers working or interested in digital health, PPIE members, technology experts, and clinicians (Table 1). Panelists reported experience with ADHD across a range of areas including lived experience, psychoeducation, and pharmacological support. All collaborators except 1 (United States) resided in European countries, mostly in the United Kingdom. All PPIE members were UK residents and English speakers. All collaborators except 2 reported experiences with digital health technologies, with most reporting 1 to 5 years of experience, and many reported 6 to 10 years of experience. Types of digital technology participants were familiar with included telehealth, mobile health, information technology, digital therapeutics, and wellness apps. Detailed characteristics of the modified Delphi panel are presented in Table 1 for rounds 1 and 2.

**Table 1.** Main characteristics of the modified Delphi panel.

Characteristics	First round (N=28), n (%)	Second round (N=27), n (%)
Location		
Belgium	1 (4)	—^a^
Germany	4 (14)	4 (15)
Ireland	1 (4)	1 (4)
Italy	1 (4)	1 (4)
Norway	1 (4)	1 (4)
Sweden	1 (4)	1 (4)
United Kingdom	18 (64)	18 (67)
United States	1 (4)	1 (4)
Experience with ADHD^b^		
Digital health technology	23 (82)	23 (85)
Have lived experiences with ADHD	10 (36)	10 (37)
Psychoeducation for ADHD	19 (68)	19 (70)
Psychosocial or nonpharmacological support for ADHD	19 (68)	19 (70)
Pharmacological or medical support for ADHD	11 (39)	11 (41)
Other areas of ADHD research	16 (57)	15 (56)
Other	4 (14)	4 (15)
Years of experience with ADHD		
1‐5	4 (14)	3 (11)
6‐10	4 (14)	4 (15)
>10	19 (68)	19 (70)
Not reported	1 (4)	1 (4)
Experience of digital health technologies, n (%)		
None	1 (4)	1 (4)
Digital therapeutics	13 (46)	12 (44)
Health information technology	6 (21)	6 (22)
Mobile health	18 (64)	18 (67)
Telehealth	7 (25)	7 (26)
Wellness apps	13 (46)	13 (48)
Other	2 (7)	2 (7)
Years of experience with digital health technologies		
<1	4 (14)	3 (11)
1‐5	15 (53)	15 (56)
6‐10	7 (25)	7 (26)
>10	1 (4)	1 (4)
Not reported	1 (4)	1 (4)

a Not applicable.

b ADHD: attention-deficit/hyperactivity disorder.

### Round 1 Survey

#### Initial Statements

Our first EUNETHYDIS SIG meeting included a discussion among SIG members to agree on the overarching statement areas and topics to consider. These were translated into the initial statements that went into the Delphi (Multimedia Appendix 2) by TN-D, AP, and EF, based also on review of the literature and other consensus statements on digital mental health. The initial statements were organized into four sections: (1) Overall Opportunities, (2) Development, (3) Evaluation, and (4) Implementation. All 28 participants provided responses for all 19 statements. A high level of agreement was achieved for all statements within each section.

#### Overall Opportunities

Four statements about opportunities offered by digital health technologies addressed timely access to evidence-based support, expanding reach, enhancing flexibility and inclusivity, and providing just-in-time data. Agreement ranged from 89.3% (25/28) to 100% (28/28). While participants agreed that digital health technologies could improve support for ADHD, comments in the survey and during the discussion meeting raised cautions that these potential outcomes are yet to be seen. In addition, suggestions were offered to include statements about risks, alongside opportunities. These informed statement revisions to (1) use words and phrases that indicate a level of uncertainty (eg, “could” rather than “can”) and (2) generate a set of statements about risks.

#### Development

Four statements about key considerations for developing digital health technology focused on ways to accelerate product development, ensure user-centered design, promote multidisciplinary collaboration, and align digital health with existing norms, practices, and standards. The agreement ranged from 96.4% (27/28) to 100% (28/28). Most agreed on the content but suggested using fewer abstract words and splitting long statements into shorter ones to improve clarity.

#### Evaluation

Six statements about considerations for evaluating digital health products focused on unique, untested challenges given the rapidly developing field. These included statements about possibly needing flexibility in the evidence type and level required, emphasizing real-world usability and impact, and potential adverse effects, especially considering ADHD characteristics. Agreement ranged from 78.6% (22/28) to 100% (28/28), with the lowest being on the statement about evidence flexibility. Concerns were raised about the potential harms of the many available digital health products that have never been rigorously tested, especially given the likely challenges with sustained engagement among individuals with ADHD. In discussing the issue, participants seemed to agree that rigorous evaluation is needed, while the standards for—and the practical guidelines on—achieving the rigor are to be established.

#### Implementation

Five statements about the implementation of digital health technology included the need for its integration with comprehensive support for ADHD, multidisciplinary and community-participatory strategies, transparency, and sustainability. Agreement ranged from 92.9% (26/28) to 100% (28/28), and suggestions were made to further emphasize the user-centered approach in all settings, and monitoring and evaluating the implementation.

### Round 2 Survey

The statements were revised based on the feedback from the first-round survey (Multimedia Appendix 2) and discussion. Even though the target agreement levels had already been reached, we felt it was important to incorporate participants’ feedback to ensure the statements captured the important nuances identified and achieve consensus on the refined statements. The revisions therefore largely consisted of splitting existing statements or the addition of caveats as raised in the discussions. The revision resulted in 30 statements across four sections: (1) Opportunities and Aspirations, (2) Development and Evaluation (Parts 1 and 2), (3) Implementation, and (4) Risks and Unintended Consequences. For each section, qualifying statements were added to provide context. All 27 participants provided responses for the statements 1 through 22, and 26 participants provided responses for the statements 23 through 30.

The statements are presented in Textbox 1. These include minor phrasing changes made after the round 2 survey, which were shared during the second discussion meeting. Of the 30, 1 statement (13) reached 92.6% (25/27), 2 statements (27 and 29) reached 96.2% (25/26), 4 statements (4, 10, 12, 14) reached 96.3% (26/27) agreement, and the remaining statements (n=23) reached 100% (27/27) agreement, indicating an even higher level of agreement across statements compared to round 1.

Remaining concerns from the panel focused on the statements being too general and abstract, lacking concrete examples or actionable guidelines. While we acknowledge these concerns, we intended to generate consensus statements on general principles to inform further work in this rapidly developing, heterogeneous field. Given the aim, participants agreed to move forward with sending the revised set of statements to the EUNETHYDIS membership for ratification. Prompted by a PPIE member who expressed challenges in participating in the discussion meeting and questioned the practical implications of the statements, we also organized another opportunity to share and discuss the final statements among PPIE members only. In this meeting, we sought feedback about the research process and answered any outstanding questions.

### EUNETHYDIS Membership Ratification and PPIE Meeting

The final statements, together with the summary of the Delphi study, were sent to all 129 EUNETHYDIS members, 2 weeks prior to the September 2025 annual network meeting, providing opportunities to comment or ask questions by email. During the membership segment of the annual meeting, an in-person discussion was led by AP and EF, and the 52 members present voted unanimously (by a show of hands) to ratify the statements. The comments included a suggestion to provide references to empirical evidence in support of the statements, and the importance of acknowledging that while some negative consequences may be unintended, they are often predictable. A PPIE meeting (coordinated by AP) was held in October 2025, providing a chance for PPIE colleagues to ask questions and provide comments on the writing of the paper specifically. After this, the draft of this study was shared via email with panel members for final review.

## Discussion

### Principal Findings

This project represents the first overarching expert consensus on digital health care for people with ADHD, successfully producing 30 statements across 4 critical domains. A high level of agreement was achieved for the final set of statements addressing (1) Opportunities and Aspirations, (2) Development and Evaluation, (3) Implementation, and (4) Risks and Unintended Consequences of digital health care for individuals with ADHD. This demonstrates strong alignment among diverse stakeholders about key considerations for ADHD digital health development, despite the heterogeneous nature of the field. Our findings align with broader digital mental health guidance [13-16] while highlighting ADHD-specific considerations. Our statements are highly nuanced, balancing the potential opportunities and risks and reflecting the experiences of diverse expert panel members, including PPIE collaborators. This work addresses previous concerns that the perspectives of those with ADHD have been neglected when exploring digital health innovations [41].

The agreement among panel members on the 2 rounds of the Delphi survey was high, indicating stability in the consensus. Although more than 75% (78.6%, 22/28) of the participants agreed on the initial draft statements, revisions were made to incorporate comments received and feedback from discussions during the online meeting. This was important in establishing consensus as the statements were designed to be general and thus relatively easy to agree with, but people still had strong opinions and important suggestions. As a result, 100% (n=27) of the round 2 participants agreed on most of the revised, final statements, and the EUNETHYDIS membership supported them unanimously.

To incorporate the feedback, the number of statements was increased from 19 to 30 in the revision, and the section on risks and unintended consequences was added. This reflects our effort to capture the important nuances identified by the panel members, ensure each statement had 1 clear point, and that possible risks were sufficiently emphasized together with opportunities. Engagement activities consistently emphasized attention to the words used, and to how specific terms were interpreted. Subsequently, language was carefully reviewed to ensure clarity and express appropriate levels of uncertainty, as many points in the statements remain to be tested. We feel that the iterative development resulted in the consensus statements that are detailed, nuanced, and clear.

Challenges associated with evaluating digital health products were discussed extensively, and this was reflected in the final statements. This is likely partly due to a high proportion of researchers represented in the expert panel. Some participants emphasized the importance of the quality assurances (eg, through rigorous clinical trials, evidence synthesis), and others pointed out the challenges of applying the traditional methodologies to evaluating rapid and iterative digital health innovations. Evaluation procedures, such as comparing outcomes between experimental conditions and blinding users to these conditions, may be particularly difficult with digital interventions. Effectiveness has not been routinely measured in some areas of digital innovation. Participants with lived experience emphasized the importance of measuring outcomes that matter to them, consistent with recent literature and movements emphasizing the importance of real-life outcomes beyond symptom reduction or technical metrics [42,43]. There was agreement across the panel, however, that appropriate evaluation methods and standards must be established. It was also agreed that better and user-friendly methods are needed to identify and communicate the quality of the products offered, so that this is clear to clinicians, researchers, and those with lived experience. A PPIE member shared the experience of signing up to multiple wellness apps at night and not being able to follow through with any, illustrating the real-world cost of insufficient quality control.

The statements were intentionally general to establish consensus among experts with diverse backgrounds and interests. During the discussions, however, the need for more specific guidelines (eg, for complex systems), practical solutions (eg, for reducing risks), and real-world examples (eg, with screen time) became increasingly apparent. Participants agreed that while these consensus statements are important, having them does not necessarily mean they will be applied in practice, highlighting the importance of continued work to develop more tailored guidelines and conduct high-quality research. In particular, work is needed to clarify how and whether the general consensus principles apply differentially to varying classifications and risk tiers of digital health products, and to develop and test practical approaches. This work will be taken forward by the EUNETHYDIS Special Interest Group on Digital Health.

### Strengths

The coproduction process, including PPIE members, allowed us to incorporate important nuances and emphasize a user-centered approach. The close working relationship with a recognized group of ADHD researchers, EUNETHYDIS, has helped us gather highly qualified professional experts and increases the likelihood of the consensus statements being considered seriously and representing an authoritative body of expertise. Experts in other areas were identified and invited systematically to ensure diverse stakeholder involvement. While other strengths include attention to qualitative participant feedback, the composition of the expert panel was likely the most critical element contributing to the successful establishment of consensus.

### Limitations

The expert panel members who participated in the Delphi survey had strong interests in digital health care for people with ADHD. This ensured a high engagement level but may have excluded the voices of those more skeptical of the role of digital technologies. However, there were many discussions on risks and unintended consequences and need for quality control, suggesting cautious attitudes toward digital health innovations among panel members [44]. Additionally, the consensus statement was approved unanimously by the 52 EUNETHYDIS members in attendance at the annual meeting (and shared with all 129 members by email), including individuals who were not part of the Digital SIG, reflecting wider consensus from ADHD experts.

We acknowledge that our use of live, nonanonymous discussion meetings between survey rounds required us to ensure these discussions did not promote “groupthink” or marginalize any voices or perspectives and that the coordinating team (AP, EF, TN-D) had a dual role as panel members, which could potentially introduce bias. We addressed this through setting specific ground rules to frame the discussion, and ensure no voice was privileged over another. This was a collaborative and engaged piece of work, corresponding to UK public involvement standards [34]. With 36% (10/28) of our panel having lived experience of ADHD, we ensured that their voices were not marginalized but well represented in our discussions. A gap of several weeks between discussion meeting and subsequent voting also mitigated the effect of any groupthink bias.

This consensus statement aimed to establish agreement, and while we made efforts to consider disagreements and take them into account during discussions and when revising the statements, we did not explicitly analyze disagreements or outlier opinions. The exploration of disagreement and outlier responses can help identify if experts (eg, individuals working in industry compared to those in academia) have distinct views that could provide additional context to the consensus statement [44].

There are also important areas that the consensus statements do not cover, such as challenges associated with screen time for youth with ADHD, risks of impulsive purchasing of digital products linked to financial vulnerability, and the use of artificial intelligence (AI). This was partly because of the stated aim of focusing on building consensus on broader topics. In relation to the rapidly growing area of AI, however, this was because, despite some mentions of chatbots and apps, the idea of including this as a topic was not raised until round 2, when it was too late to take action (see Multimedia Appendix 2 for comments on AI from rounds 1 and 2).

In addition, the engagement of PPIE members could have included more discussion opportunities. The PPIE members were all UK residents, partly due to the statements and Delphi survey being in English. Lived experiences of people from other European countries may be different, and the inclusion of them could have offered different perspectives. Similarly, two-thirds of the overall panel were from the United Kingdom, which we acknowledge may impact how these findings may be generalized and applied across diverse health care systems, cultural contexts, and languages in Europe. We recommend that these broad consensus statements subsequently form the basis for more context-specific implementation frameworks, with scope to explore more topics in further detail. We also recommend that the integration of AI into digital health care for people with ADHD is included as a theme in future work.

### Implications

This European expert consensus provides the first comprehensive set of key considerations for digital health care for people with ADHD. The successful development of 30 consensus statements through an iterative Delphi process demonstrates the feasibility of achieving expert agreement on complex, rapidly evolving topics, such as digital health. The consensus statements offer a balanced framework that embraces innovation opportunities while maintaining focus on research evidence, safety, and equity considerations. We hope that the statements provide a foundation for coordinated, evidence-based development and research of the next generation of digital health care tools, which must meet the diverse needs of people with ADHD and their families. Regular review and updating of these consensus statements will be essential to keep pace with the evolving digital health landscape. Future work should focus on translating these considerations into practical implementation frameworks within specific areas, identifying priorities, and connecting them to real-life stories and empirical evidence. Together with emerging research, this consensus suggests that digital technologies have the potential to support ADHD management. However, coordinated, multidisciplinary, coproduction approaches will be essential to address current limitations and to accelerate development, ensure rigorous evaluation, and support the sustainable integration of digital technologies into routine ADHD care.

## Supplementary material

10.2196/85638 Multimedia Appendix 1 Summary provided to panel members.

10.2196/85638 Multimedia Appendix 2 Statements and feedback received (some feedback comments have been edited to maintain confidentiality).

## Abbreviations

ADHD: attention-deficit/hyperactivity disorder
AI: artificial intelligence
EUNETHYDIS: European Network for ADHD
PPIE: public and patient involvement and engagement
SIG: Special Interest Group
