# Supplementary material for: Consensus Statement on Digital Health and Attention-Deficit/Hyperactivity Disorder by the European Network for ADHD (EUNETHYDIS): Modified Delphi Study
Source: J Med Internet Res. 2026 Jul 16;28:e85638. doi: 10.2196/85638 (PMC13374793; doi:10.2196/85638)
Supplement: Multimedia Appendix 1 [file jmir-v28-e85638-s001.pdf]

Appendix 1. Summary provided to panel members.

Consensus statement on digital health in ADHD

Background

Digital health technologies are becoming an important part of healthcare, including for people with ADHD. However, creating and using these technologies effectively is challenging. People with ADHD may struggle with focus, organization, and communication, which affects how they engage with digital tools. ADHD also varies widely between individuals, making it hard to design solutions that work for everyone. At the same time, digital health has great potential to help people with ADHD, offering flexible and innovative support tailored to their needs.

Aims

To advance research in this area, a group of European ADHD experts, technology specialists, and people with lived experience are coming together to agree on key priorities and considerations for developing, evaluating, and implementing digital technology for ADHD.

Methods

Key statements will be drafted, building on discussions held during a European ADHD Network (Eunethydis) Special Interest Group (SIG) meeting. An Expert Panel will be convened, to include key ADHD researchers, technology experts, and people with lived experience of ADHD. Statements will be shared with Panel members via a Delphi survey (likely two rounds) to gather insights from multiple perspectives. Panel members will work together to refine and prioritise statements and build consensus on this topic.

Dissemination

Final recommendations will be written up for publication in a peer reviewed journal (TBC) and shared with the wider ADHD research community, with the aim of helping to shape innovations in ADHD digital health.

Practical Information

- **Organisers:** Dr Anna Price, Dr Emi Furukawa, and Dr Tamsin Newlove-Delgado on behalf of the European ADHD Network (Eunethydis) Digital Health Special Interest Group.

- **Project remit:** To produce a 'consensus statement' on ADHD and digital health; identifying priorities for developing digital health technologies for people with ADHD. This will be co-produced by researchers with a special interest in ADHD, experts in technology and experts by experience. The intention is to include and name expert panel members as co-authors (or contributors) on the consensus statement.

- **What does the work involve?** Responding to two rounds of an online Delphi survey. Reviewing and commenting on statements. Attending one or two online meetings to discuss feedback, (if available). Reviewing and commenting on the final paper as a co-author or agreeing to have your name included as a contributor.

- **Timeline:** April, Delphi 1. Meeting 1. June, Statement revision. Delphi 2. July, Meeting 2. August, draft publication. Circulate statements and draft publication with Eunethydis membership for review ahead of September 2025 Annual Meeting.
