# Supplementary material for: Consensus Statement on Digital Health and Attention-Deficit/Hyperactivity Disorder by the European Network for ADHD (EUNETHYDIS): Modified Delphi Study
Source: J Med Internet Res. 2026 Jul 16;28:e85638. doi: 10.2196/85638 (PMC13374793; doi:10.2196/85638)
Supplement: Multimedia Appendix 2 [file jmir-v28-e85638-s002.docx]

# Appendix 2. Statements and Feedback received (Some feedback comments have been edited to maintain confidentiality)

# Round 1

(% agreement in parentheses, panel comments in text box)

## **1. Digital technology can expand timely access to the assessment of and evidence-based support for ADHD characteristics and related challenges** (100% agreement)

- I think digital technology can be very useful to speed up access to ADHD assessments and support. Teams assessments can work very well. However in my experience some young people don't engage very well in online sessions for multiple reasons.
- Digital technology could speed up assessment for ADHD and offer more timely intervention options (just trying to unpack a rather complex statement a bit)
- Digital technology can expand and improve access to the assessment of and evidence-based support for ADHD characteristics and related challenges in a timely manner
- "Only with evidence of efficacy, effectiveness and successful implementation in routine clinical care!"
- "Would prefer ""Digital technology has the potential to expand timely access to the assessment of and evidence-based support for ADHD and related challenges"" Not sure that the characteristics adds anything here?"
- "There is potential, but did we reach a stage where we developed evidence-based technologies that are rigorous enough to be used in clinical settings? An important question to reflect upon, at least in my opinion."
- "It may be helpful to separate out assessment and support in this statement. I've not seen a good digital assessment of ADHD yet, but I have seen a number of effective supports for the condition."
- I think there are two separate issues here: 1. Digital technologies could improve timely access to the assessment of ADHD-related difficulties, and 2. Digital technology could enhance the provision of evidence-based interventions for ADHD and associated challenges.
- Possibly worth segregating assessment and evidence-based support as two different aspects of treatment

## 2. Digital technology can extend the reach of high quality support to more people, especially in regions with limited mental health resources (100% agreement)

- Maybe Not “high quality” but rather “better”
- Depends on what digital technology and how easy it is to use and personalised it is. My experience as a person with ADHD of using apps is that I forget I’ve got them and the novelty wears off quickly.
- It can be very difficult to get to face to face appointments especially if the person lives outside of a town. People (…) also don't often have access to a car.
- “Could” rather than “can”? Not sure it does yet!
- But needs to be evaluated and evidenced bases for this more marginalised populations
- I wondered about 'has the potential to' but this is probably too wordy. On reflection I wonder if a qualifier is needed about not being used to replace some interactions or elements of healthcare (i.e. lower quality substitution) but that may be a different discussion and statement
- "Definitely, once again it would be important to reflect how are benefits and challenges balanced. For example, in LMICs do we want technology to substitute clinical professionals?"
- "This is absolutely one of the key reasons to develop the technology. There's a real opportunity here to improve quality of care even where there is existing provision. There are far too many people in healthcare and beyond who are asked to support those with ADHD, yet have not had adequate training to do so. Technology can support them in engaging according to a good evidence base."
- "Digital technology can extend/help to facilitate the delivery of high-quality psychological support to people who need it, particularly in underserved regions (e.g., limited coverage of in person clinical services) or populations (e.g., minority groups, people in full-time work, non-English speakers). "
- "As my experience is that there is limited access to mental health resources globally, I would consider removing the ""especially in regions with limited mental health resources."" I would consider adding something along the lines of "" Digital technology can extend the reach of high quality support to more people, especially given the global issue of limited mental health resources."
- Could be worth clarifying the limited mental health resources (eg. Waitlists affecting services vs countries with limited services)

## 3. Digital technology can enhance flexibility and inclusivity by accommodating diverse preferences for content, access, language, stimulus types, and interactions (96.4% agreement)

- Digital technology can enhance flexibility and inclusivity by accommodating diverse and personalized preferences for content, access, language, stimulus types, and interactions.
- I am not sure how this would work. Digital interventions can be more flexible compared to face-to-face interventions. I am less clear how it can support preferences for content. When we say interactions, who do we anticipate the interactions are with, people, AI chat bots or something else?
- Yes, tech can help with this. Especially delivering information in different formats..
- The statement is true in that it can, but it's not a given that it will. Many existing implementations have the opposite effect. It may be better to revise the statement to make that clear.
- It may be good to consider whether this should be framed in the context of of accessibility (e.g., variety of formats, language), user-centred design (focus on individual preferences-making it as user-friendly as possible), or equitable/inclusive access (accommodating diverse language, culture, neurodiversity, other individual differences, etc). I think all contexts are important but require a slightly different framing.

## 4. Digital technology can provide just-in-time data to improve monitoring, feedback, and personalized support (89.3% agreement)

- I think that AI apps that people can talk to in the moment of distress can be very helpful. (…) for me remembering they are there is the challenge.
- Not sure about this. Monitoring sounds intrusive and invasive. Rather “Big Brother is Watching You”.
- It is less clear to me whether that is what end users want, and in the context of neurodiversity endless real time feedback could become confusing, distracting or feel like a demand "
- "I've answered 'agree' rather than 'strongly agree' to all these statements because the evidence isn't there yet, it is still 'have the potential to' rather than 'can'"
- "Yes, once again - though - I do not think we are still there, in terms of evidence-based tools which are clinically usable."
- "It could definitely in theory, but I've yet to see it do so. Doing this in any meaningful way is likely to be challenging given the unique personalised nature of each person's support needs."
- "Is it feedback for clinicians, patients, families, etc?"
- This seems like it could be a tricky subject given the on-going conversations around data privacy.

## 5. Accelerate digital health product development through knowledge and resource sharing among researchers, clinicians, and people with lived experience, with increased support from institutions and funders (100% agreement)

- It is so important that clinicians and researchers in this field are ADHD themselves or have people in their team that are. I am a clinician with ADHD, and this helps me connect with the young people I work with on a level that others can't. I find I can use the right language and share examples of shared experience that gains trust. People with ADHD are used to feeling like people don't get them and if they sense this off an app, a digital system or a researcher then the likelihood is they will shut down or mask.
- Co-production is always the way forwards I think
- This is needed. It is also needed that industry partners collaborate with researchers by funding their pilot projects which creates the evidence base for new technology and not only ask researchers (and fund them) to ""test"" a new technology, especially when such tech was not developed based on solid evidence.
- The message in this sentence is not completely clear to me
- It's vital all stakeholders are represented in this process. Ideally, I'd extend ""resource sharing"" to include open research and open-source development practices, so that the widest group possible can contribute. See AutSPACES as an example - <https://www.turing.ac.uk/research/research-projects/citizen-science-platform-autistica>
- I wonder if the statement about institutional commitment and funding support could be separated.
- The ‘accelerate’ aspect of this statement is slightly confusing.

## 6. Ensure user-centered design by involving diverse stakeholders early, balancing researcher and user priorities, and integrating continuous feedback (100% agreement)

- Strongly agree with this as per my previous comment. Nobody understands an ADHD brain like a person who has it. I still find it astounding how much shared experience I have with all of my fellow ADHD friends, colleagues and patients. However each though many things are shared it is a spectrum and the profile is often spiky. This needs to be taken into account. You can't develop something with only a few ADHD voices as it won't serve all.
- Important to listen to ALL voices, not only most vocal ones though.
- User-centredness is less around priorities and more around ontological positioning. It's vital to solve problems in a way that fits the way that users experience them. Until this point, many of the interventions have failed because they failed to understand the whole problem space from the end-user perspective and have therefore not been workable when used in the real world.
- This statement is super broad, e.g., it is not clear who might be these 'diverse stakeholders' and what kind of priorities should be balanced. Perhaps the plan is to expand on this in the text, in which case, please ignore this comment.
- I would include clinicians in this statement.

## 7. Foster multidisciplinary integration of field-specific perspectives, expertise, languages, platforms and methodologies (96.4% agreement)

- I am disagreeing with this, but to be honest I am not sure I understand it! Does the expertise include lived experience, if so, how is this different from the first option?
- Agreed, however I feel this is already the case in the field of ADHD, where we naturally conduct multidisciplinary collaborations and research
- The meaning of this sentence is not completely clear to me (e.g., what does 'multidisciplinary' and 'field-specific' mean in practice?)
- Absolutely - tooling and practices should be aligned to support this as much as possible, too.
- I think this statement could be broken down into two: 1. Focus on communication/collaboration, e.g., Foster the integration of field-specific perspectives, expertise, and languages to support/enhance interdisciplinary collaboration and communication. 2. Focus on methods: Adopt/use various platforms and methods to ensure effective and efficient approaches to address the existing challenges (e.g., research and clinical).

## 8. Align digital product design with community norms and cultural practices, as well as clinical and privacy standards (96.4% agreement)

- A digital product has to align with life. If something feels like it is too clinical and not understanding or wanting to understand me as an individual, then I am turned off from that product.
- I think this would be a disaster, what are the cultural norms in this area for a set of disorders that are not well understood by the general public.
- Yes, definitely, technologies developed in a specific country or directed to a specific audience, may not be well received in other countries or by other groups
- Can you clarify what you mean by 'community norms and cultural practices' in reality? I have indicated 'agree' but it's hard for me to answer this one, as it's not fully clear to mean what the sentence means in more practical terms
- Any development of solutions should be culturally aware and fit into the general life of its users. However, it's worth noting that the conceptualisation of ADHD and the way it manifests is at least somewhat culturally specific to the Western European, particularly anglophone context.
- Maybe add something about how this alignment occurs and/or why is it important?

## 9. Allow flexibility in the required level of evidence depending on product types, from digital resources supplementing existing care to novel intervention (78.6% agreement)

- We just don't have good and reliable ADHD evidence yet. Much of the evidence is still gender biased. Much of the evidence-based treatments for ADHD have not for some reason been translated into clinical practice yet. New research needs to be funded to allow innovation in the field.
- Not sure how the term flexibility can be interpreted within this context. I would need more clarification before agreeing.
- We don't have the time or access to the funding unfortunately to run everything through an RCT design
- this might need a bit more clarity -especially the second half of the sentence? may also need qualifying that we would still expect rigorous evaluation
- There is the NICE Evidence Standards Framework which specifies some standards for evaluating digital health products.
- This is what probably caused the ongoing situations, where there are tons of tools and apps, most of which very low quality, not evidence based, and not helpful clinically.
- Difference approaches will necessitate different forms of evidence. Interventions for ADHD have been historically weak in that area, often relying on report from third parties about perceptions of behaviour.
- Should there be some consideration for the level of evidence being proportionate to the product type?
- I would consider adding to the statement - with continual review of data to support the highest level of care.
- It may be helpful to have a couple of examples, such as this may mean more evidence required for direct intervention through digital technology that for presenting several behavioural options or techniques that people can choose to try.
- we should always aim at the highest level of evidence

## 10. Assess usability, feasibility, efficiency and effectiveness while incorporating large-scale, continuous real-world feedback (100% agreement)

- Agree, however continuous real-world feedback need to be defined/operationalzed earlier
- My only concern here is that effectiveness is at the end of that statement rather than at the beginning
- Yes. Also, at early stages we should have consultation with people from "the real world"
- What would 'large-scale continuous real-world feedback' mean in practice?
- This sounds like integrating evidence-based working with a CI/CD development model, which would be a very valuable practice in many fields.
- Sounds great!
- I think this largely depends on what the "thing" is, there can be times when looking at developing additional capabilities to a digital intervention, where that capability may be optional for an end user, that this process if fully applied means the additional capability never gets built.

## 11. Evaluate inclusivity, reach, and impact on intended and unintended users (96.4% agreement)

- People with ADHD are impulsive and sign up to anything in moments of sadness or desperation. I would like to see usage tracked and evaluated and then refunds given if they haven't used the system. It is so important to consider the financial vulnerability of people with ADHD.
- What would be value be of comparing against unintended users. Also, why would unintended users even engage?
- For sure this is important, however before thinking of "unintended" users, it is important to make sure we have adequately supported "intended" users, i.e., people with ADHD
- Perhaps change 'intended' to 'target users'?

## 12. Select objective and subjective outcome measures that leverage digital environment while considering and minimizing user burden (96.4% agreement)

- “Leverage digital environment” is a bit unclear. If it means what I think it means I agree!
- I think reducing user burden will be important, and micro trials might be helpful here
- Important to include some "subjective" outcomes, however we need to be very careful, since we need to develop evidence base tools, so such evidence needs to be built on objective outcomes first
- What does 'leveraging digital environment' mean in practice? Overall, my feedback would be that there are lots of 'big' words where it sometimes not fully clear what this would translate into in practice. It would be great to try to write in a more accessible, explicit way and give examples of what you mean
- Truly objective measures in this area are incredibly hard to come by, so should be approached with extreme care. Objectivity has historically been a concept used to marginalised lived experience, particularly where it differs from medical/academic models.
- Identify and select?

## 13. Identify unintended challenges and adverse effects, considering ADHD characteristics affecting attention, learning, motivation, and technology dependence (100% agreement)

- Perhaps: “Identify unintended challenges and adverse effects that might be posed by ADHD characteristics affecting attention, learning, motivation, and technology dependence.”
- It will be important to demonstrate any side effects of digital interventions
- Yes, definitely important, especially in children and young people
- Definitely need to explore adverse effects, but I'm not clear what the end of this phrase means. Does it mean considering that ADHDers may be particularly vulnerable to downsides of digital intervention?
- Consider and identify?
- Possibly include lived experience of negative consequences, e.g., unintended stigmatisation

## 14. Report on user involvement, study design justification, and all clinically-meaningful outcomes including unintended consequences (100% agreement)

- Perhaps begin: Researchers should report…
- Maybe not just clinically meaningful but also system outcomes (i.e. could this increase contacts in one part of the system, will there be unintended consequences for health or other services)
- Include measures of engagement with the technology as well
- Yes, this should be the norm to produce high quality tools
- There's lots of different things here in a single sentence. And I wonder what 'all' clinically- meaningful outcomes means in practice. Also, some overlap with what has been in some previous statements.
- Definitely - full transparency is vital. No intervention is a panacea, and so it's important to give people all the information possible to make an informed decision about level and manner of use.

## 15. Integrate digital technology into comprehensive support for ADHD, recognizing its clinical, technical and equity limitations (100% agreement)

- As long as we are confident it will be helpful!
- The evidence isn't there yet
- Okay. But "what" technology? There are already multiple techs available. As experts, we must tell people which are good/helpful, and which are not.
- It is not quite clear to me what 'recognizing its clinical, technical and equity limitations' means in practice

## 16. Employ multi-disciplinary and community-participatory collaborative care strategies, especially in non-traditional care settings (e.g., schools, community hubs, places of worship) (92.9% agreement)

- I guess but this doesn't preclude sign posting from more traditional routes as well such as primary care
- Great - linking to the NHS social prescribing model which talks about harnessing community assets/spaces might be useful.
- I am not sure why the non-traditional care settings is emphasised here.
- Definitely, we need to honour in the real world while developing new techs
- Yes - though I'd like to explicitly include the value of peer support in this. Such communities can be vital connection points for future research efforts too.

## 17. Take responsibility in educating users about digital health products, their quality, and how to select suitable options (96.4% agreement)

- Again very good question - links to Darzi reports recommendation about digital literacy and confidence for our populations as well as staff in directing to these tools.
- we might need to say who should take responsibility?
- I am not sure this is a priority.
- To the extent that this is informing rather than convincing, and giving people what they need to make the best decision in their own circumstances. There is a risk here that interventions may become effectively mandated by public care and support pathways, and so it is vital to be clear about when an intervention is not useful, or actively harmful.

## 18. Ensure transparency about data collection, privacy protections, and product limitations (100% agreement)

- And the IG processes maybe?
- Yes, if possible, make everything open access and transparent. This should also be clear when working with industry partners. Financial conflicts of interest are seen very badly sometimes
- Transparency and as far as possible, control. As far as possible, facilitating personal data sovereignty.

## 19. Build in sustainable implementation strategies beyond the scope of research, such as industry partnerships, economic evaluations, and iterative development plans (96.4% agreement)

- “Sustainable” could be a bit ambiguous here given its widespread use in the ecological sense. Something like “Build in implementation strategies that will ensure a robust lifetime for the product” or something better.
- I think economic evaluations will be important, there are on-going costs to maintaining and developing digital interventions, so they must be cost effective to justify those costs
- and ongoing capacity/resource?
- not sure about 'beyond the scope of research' here - could cut?
- Yes, this is why we need a strong focus on evidence base, since we want to provide clear recommendations to policy makers and produce clear guidelines, so understanding economic impact would be crucial
- To the extent that it is the effectiveness of the intervention that leads, rather than the profitability of an intervention.

## General feedback (Round 1)

- Looking good!
- "As the survey is being completed by the Digital SIG, I wonder if this will lead to a skewed picture. It would be good to invite the whole Eunethydis community, including those not working/interested in digital technologies."
- Sounds very comprehensive! Thank you.
- Implementation section could include statement regarding the importance of centring human-design or ensure people with ADHD are consulted in terms how to best implement technology

# Round 2

**Section 1. Opportunities and Aspirations**

## 1. Digital technologies could improve timely access to assessment and evidence-based support for ADHD and related challenges. (100% agreement)

## 2. Digital technologies could enhance the flexibility and inclusivity of services by accommodating diverse preferences for content, access, language, stimulus types, and interactions (100% agreement)

## 3. Digital technology could extend the reach of high quality support for ADHD, especially given widespread challenges of limited mental health resources across countries with differing health infrastructure (100% agreement)

## 4. Digital technology has the potential to provide just-in-time data to improve monitoring, feedback, and personalized support, as long as it is user-centred, and addresses privacy concerns (96.3% agreement)

- I don't disagree per se - just some tweaking to make the language more tentative: “Digital technology has the potential to provide just-in-time data that supports improved monitoring, feedback, and personalised support. However, its success depends on being user-centred, accessible, and attentive to privacy concerns (or careful about addressing privacy concerns).”

## 5. Opportunities for improved provision must be viewed in the context of a range of risks and unintended consequences including, but not limited to, unregulated digital development, inappropriate substitution for clinical care, and widening of inequalities (see Section 4) (100% agreement)6. Ensure user-centered design by involving diverse stakeholders early, balancing researcher and user priorities, and integrating continuous feedback (100% agreement)

**Section 2: Development and Evaluation**

***Section 2a: Development***

## 6. Digital health product development could be improved through better knowledge and resource sharing among researchers, clinicians, software engineers, and people with lived experience (100% agreement)

## 7. User-centred development is key to ensure technologies are effective, by involving diverse stakeholders early, balancing researcher, clinician, and user priorities, and integrating continuous feedback (100% agreement)

## 8. Researchers need to adopt multidisciplinary ways of working to ensure development integrates different perspectives, varied expertise, and a range of digital platforms and methodologies (100% agreement)

## 9. Development of evidence-based digital technologies for ADHD requires support from institutions and funders to create and resource diverse, user-centred and multidisciplinary teams (100% agreement)

## 10. Digital product design needs to be sensitive to culture and context, as well as meeting clinical and privacy standards (96.3% agreement)

- 'Clinical standards' may be too vague on its own: how about more detailed description, e.g., established clinical standards for safety, efficacy, and evidence-based practice?

***Section 2b: Evaluation***

## 11. Digital health products should undergo rigorous evaluation in order to ensure their benefits and avoid harm (100% agreement)

## 12. Guidelines and methodologies are needed so that researchers can establish a reliable and robust evidence-base, in the context of the rapid and iterative development of ADHD health technologies (96.3% agreement)

- How about a slight rephrasing? Something along the lines: "To support the rapid and iterative development of ADHD health technologies, clear guidelines and innovative and agile methods are essential for establishing a reliable and robust evidence base.”

## 13. The development of new technology itself offers a potential opportunity for different forms of intervention evaluation (92.6% agreement)

- “The development of new technologies offers opportunities for novel approaches to intervention evaluation, including real-time data collection, iterative testing within co-development cycles, or other novel approaches that extend beyond traditional research methodologies” (or something along those lines).

## 14. It is important to assess effectiveness, usability, feasibility, and efficiency while incorporating large-scale, continuous real-world feedback (96.3% agreement)

- Also something about their cost-effectiveness and affordability?

## 15. Evaluations must address inclusivity, reach, and impact on intended and unintended users (100% agreement)

## 16. Objective and subjective outcome measures should be selected that are appropriate for use in digital environments, while considering and minimizing user burden (100% agreement)

## 17. Adverse effects must be identified and evaluated, including consideration of the ways ADHD characteristics can impact attention, learning, motivation, and technology dependence (100% agreement)

## 18. Research should report on user involvement and engagement, study design justification, and clinically-meaningful outcomes including risks and unintended consequences. (see Section 4 below) (100% agreement)

**Section 3: Implementation**

## 19. Integrate digital technology into comprehensive support for ADHD whilst recognizing its clinical, technical, evidence and equity limitations (100% agreement)

## 20. Employ multi-disciplinary and community-participatory strategies, to make sure digital technologies are usable and useful in real-world settings (100% agreement)

## 21. Those implementing digital technologies need to take responsibility for educating users about digital health products, their quality, and how to select suitable options (100% agreement)

## 22. Clinicians working with people with ADHD require training and support to ensure they have the necessary competences to use digital health technologies in their practice (100% agreement)

## 23. Health and care services using digital technologies must have appropriate infrastructure and technical support in place to support end-users, protect privacy, and avoid increasing the burden on clinicians or people with ADHD (100% agreement)

## 24. Those implementing digital technologies must ensure transparency about data collection, privacy protections, and product limitations (100% agreement)

## 25. There is a need for researchers, and developers to consider sustainable implementation strategies including industry partnership, conducting economic evaluations, and planning for iterative development (100% agreement)

**Section 4. Risks and Unintended consequences**

## 26. Digital health technologies for ADHD may be used in a range of ways and contexts, and therefore the risks and unintended consequences may be unpredictable, and specific to the user/s and the system (100% agreement)

## 27. There are obvious risks relating to widening inequalities due to digital exclusion and other factors, and ways to monitor and mitigate this risk require further consideration (96.2% agreement)

## 28. Care must remain person-centred. It must be recognised that not everyone benefits from or prefers digital tools, and that some individuals with ADHD or with co-existing conditions may face challenges in interacting with digital technology (100% agreement)

## 29. The use of digital technologies with children with ADHD and their families may also require specific consideration around issues and potential risks such as consent, privacy, and screen time (96.2% agreement)

- Screen time in not a great construct, perhaps rephrase to recognise the issue of dependence or problematic use.

## 30. There is a risk that digital interventions may be used to replace rather than augment non-digital services, and hence we emphasise the importance of an integrated approach, and the provision of appropriate alternatives (100% agreement)

## General feedback (Round 2)

- These statements have been much improved - well done!
- Does the working group include any individuals with ADHD who are currently using digital products or have experience of using these for managing their condition, receiving psychological support? If not, it would be important to include them in the development of these statements.
- These look like a very good set of recommendations.
- I think the statements captured what we talked about. However, one aspect that has not yet been widely addressed is the use of AI in digital instruments. It would be valuable to clarify how AI should be employed—for instance, as a tool to support clinical decision-making rather than as an autonomous diagnostic agent—as well as to emphasize the need for transparency, ethical guidelines, and requirements for scientific validation. This is particularly relevant as an increasing number of digital tools integrate AI-based functionalities.
- As before the items were pretty much impossible to (dis)agree with - so I am not quite sure how useful the exercise is.
- Specifically for ADHD we need to give greater thought to how to structure and deliver digital interventions in a way which work with rather than against the individuals core ADHD symptoms and impairments. Greater consideration also needs to be given to what constitutes appropriate levels of engagement in evaluation studies of digital interventions, it may not be the same as engagement levels for face-to-face interventions
